# The Muscles of the Eye

**Published:** 1907-04

**Authors:** 


### BOOKS AND AUTHORS.

**The Muscles of the Eye.** By **Lucien Howe,** M.A., M.D., Professor of Ophthalmology, University of Buffalo, Member of the Royal College of Surgeons of England, Member of the Ophthalmologische Gesellschaft, of the Société Francaise D'Ophthalmologie, and of the Ophthalmological Society of the United Kingdom; former President, Sec. Ophthalmology, American Medical Association. In two volumes. Volume I. Anatomy and Physiology, including instruments for testing and methods of measurement. Illustrated. 8 vo. pp. xii-470. New York and London: G. P. Putnam's Sons. 1907. (Price, Cloth, $4.25).

One of the important medical and scientific literary events of the year is the publication of the first volume of the work of Dr. Lucien Howe, of Buffalo, on "The Muscles of the Eye." The subject is one which has especially engaged his attention for many years, and to which he has given much study and extended experimentation. This product of his pen, carefully prepared, is a summary record of the conclusions and labors of others in the same field, of his verification and amplification of their findings, in which is embodied also the devices, methods and results which are peculiarly his own. He has approached his task with an open mind, unbiased and unobscured by any fad or preconceived theory. Armed by a comprehensive knowledge of the works of others throughout the world, he has experimented intelligently, he has chosen his facts and written them up discriminately and judiciously, thus presenting a valuable treatise, the first part of which is the subject of this review.

This volume deals with the anatomy and physiology of the ocular muscles, with frequent and pertinent references to the relation of anatomical conditions and functional processes to pathological states and clinical considerations. In the first part, assigned to the anatomy, there are five chapters. Chapter one treats of the ocular muscles and their dissection, and describes them as to their origin, direction, insertion, both primary and secondary, size, variations, and the like, all in clear and concise terms. In the second chapter there is an equally satisfactory description of the intraocular muscles and other structures concerned in accommodation, including the ciliary muscle, ligament of Zinn, the structure of the lens, the position of the lens and the methods of determining it, the imperfections of the refractive media which influence the ciliary muscle, the clinical importance of the refractive media, and the accessory muscles of accommodation. The third chapter contains a good outline of the nerve supply of the ocular muscles, including the sympathetic, and of the arrangement of the cells in the various nuclei, together with their relations and connections. The fourth chapter is devoted to the blood supply, and chapter five to the comparative anatomy and embryology of these muscles.

Part second, the physiology of the ocular muscles is by far the larger portion of the book, and attacks some of the most complex physiological problems. In endeavoring to solve some of these, the author exhibits his genius in the devising of instruments and methods to facilitate their study, and while he does not claim to have fully and satisfactorily found answers, yet by the amalgamation, if we may use the term, of the results of his own labors and those of others, as he has done here, he has certainly come nearer to such a consummation.

Our limits will not permit a detail reference to the many subjects discussed in this highly interesting part of the book, or to the author's special contributions. It must suffice to indicate only a few of them in the most general manner. There are eleven chapters with several subdivisions, and in these are considered, first, one eye at rest, which reviews those fundamental principles relating to the rotation of the globe in different directions by the different muscles. In this connection, the author, by modifying the ordinary Javal-Schiötz ophthalmometer, found that it was possible to determine, quite exactly, the center of motion of the eye. Following this chapter is one on one eye in action, but not necessarily in motion, as in accommodation. In this also is considered the mechanicsm of accommodation, and the theories regarding it, the pupillary reactions, and the action of cycloplegics and myotics, together with the measurement of their effects in their maximum and minimum doses, by which measurements the author hopes to assist in determining whether there exists a tendency to excessive or insufficient accommodation.

The next chapter under the heading, one eye in motion, considers the manner in which the extraocular muscles move the globe, and attention is confined, first, to the motion of one eye only, eliminating all forms of associated movements, and studying the action of a single muscle. For the first time, the lifting power of the adductors and the tensile strength of the recti muscles are noted, the measurements being made by a method which is original with the author. In this chapter he also studies, quite at length, the limit of the field of fixation as determined by the tropometer and the perimeter with certain improvements that he found he could make. Due emphasis is properly given to the clinical bearings of a knowledge of this limit.

Following this is a chapter on both eyes at rest, in which the author undertakes to measure the interocular base line and to determine the position of rest of the visual axes and the position of the vertical axes. In this connection he describes what he calls physiological heterophoria and the various instruments for determining the position of rest, such as the phorometer, Maddox rod, clinoscope, and the like.

The fifth chapter takes up that group of movements which is described under the general heading, both eyes in motion. It is divided into four groups of associated movements. In the first group, the visual axes being in the primary position, the vertical axes may turn in or out. The second group of motions includes those in which the parallel visual axes move in some one of the principal meridians,—up, down, in, or out. In the third group are those movements in which the visual axes move obliquely from the primary position into some secondary position. The fourth group of associated movements, which is regarded as the most important in its clinical aspects, is studied in five subdivisions. As a preliminary step to the study of this group, the well known facts relating to ophthalmological prisms are reviewed, the meaning of the meter angle is defined, and the size of it is expressed in degrees with exactness, and a table of degrees in terms of the meter angle is constructed. There is also established the difference between the minimum and maximum fusion power, a point of no small clinical importance.

From these considerations, the author passes to a study of the variations in accommodation with a given degree of convergence (relative accommodation), illustrating it graphically, and ascertaining how the measurement of relative accommodation is made. He shows how relative accommodation is influenced by increased age. After thus discussing relative accommodation, he takes up relative convergence, describing what it is, how it is measured, and what degree of exactness is necessary for clinical purposes. Finally, he examines the true torsion which accompanies convergence and what may be called relative torsion.

After enlarging upon these subjects at considerable length, and with his characteristic exactness of definition, he decides that in the act of comfortable vision for the near point, we have to deal with three principal factors, namely, accommodation, convergence and torsion.

The conclusions at which the author finally arrives are: first, that a thorough study of the anatomy and physiology of the ocular muscles is essential to a working knowledge of their pathology; second, that more exact definitions of much of the anatomy and physiology of the ocular muscles are necessary, if we would clear up in any way our confusion of ideas concerning them; third, that we need greater uniformity in methods of examination. No matter whether a test is made of the static or dynamic condition, or whether measurements are made of the power of accommodation or of convergence or of torsion, or of any of these with reference to the other, we should agree on certain procedures to be adopted; or, if that cannot be done, then each ophthalmologist should describe his methods in his own records for his own convenience, or surely, when writing for others. Fourth, that the practitioner must make his clinical examinations much more thorough than is usual, if he wishes to obtain a sufficient number of data upon which to base a valid opinion.

Following the text proper, are three appendices. The first appendix is a bibliography for this volume and contains eight hundred and thirty-two references to articles and books on the various phases of anatomy and physiology of ocular muscles. This bibliography is of inestimable value to any one who desires to study this subject. In appendix B, the author has listed a series of questions which will indicate to the ophthalmological student some problems which are not yet solved They should act as a stimulus to the making of researches that need to be pursued in the future. Appendix B tells the reader in what libraries of the United States the important ophthalmological journals of the world may be found. The last appendix contains biographical notes with portraits of Brewster, Helmholtz, Donders, Ewald, Nagel, Le Conte, Hess and Tscherning. Finally, very complete indices render references to the text easy,—a most desirable feature.

The above gives a very inadequate view of the contents of this work. To be appreciated it should be studied. Other works have been written on the muscles of the eye, but there is none which attempts to cover the field in so complete and impartial a manner as does this one.

The book is amply illustrated, both in black and color, and in every way is a fine specimen of bookmaking. The author is to be congratulated upon the success which he has attained in giving such a full and clear presentation of a difficult and obscure subject. The profession will look forward with eager interest to the publication of the concluding volume, which is to present the pathological aspects of the subject. In its completed form this work promises to have no equal in the English language, and it will stand as an enduring monument to the painstaking labors of its author.

